# Experimental maps of DNA structure at nucleotide resolution distinguish intrinsic from protein-induced DNA deformations

**DOI:** 10.1093/nar/gky033

**Published:** 2018-01-30

**Authors:** Robert N Azad, Dana Zafiropoulos, Douglas Ober, Yining Jiang, Tsu-Pei Chiu, Jared M Sagendorf, Remo Rohs, Thomas D Tullius

**Affiliations:** 1 Department of Chemistry, Boston University, Boston, MA 02215, USA; 2 Computational Biology and Bioinformatics Program, Departments of Biological Sciences, Chemistry, Physics & Astronomy, and Computer Science, University of Southern California, Los Angeles, CA 90089, USA; 3 Program in Bioinformatics, Boston University, Boston, MA 02215, USA

## Abstract

Recognition of DNA by proteins depends on DNA sequence and structure. Often unanswered is whether the structure of naked DNA persists in a protein–DNA complex, or whether protein binding changes DNA shape. While X-ray structures of protein–DNA complexes are numerous, the structure of naked cognate DNA is seldom available experimentally. We present here an experimental and computational analysis pipeline that uses hydroxyl radical cleavage to map, at single-nucleotide resolution, DNA minor groove width, a recognition feature widely exploited by proteins. For 11 protein–DNA complexes, we compared experimental maps of naked DNA minor groove width with minor groove width measured from X-ray co-crystal structures. Seven sites had similar minor groove widths as naked DNA and when bound to protein. For four sites, part of the DNA in the complex had the same structure as naked DNA, and part changed structure upon protein binding. We compared the experimental map with minor groove patterns of DNA predicted by two computational approaches, DNAshape and ORChID2, and found good but not perfect concordance with both. This experimental approach will be useful in mapping structures of DNA sequences for which high-resolution structural data are unavailable. This approach allows probing of protein family-dependent readout mechanisms.

## INTRODUCTION

Forming a protein–DNA complex involves two molecular partners, which must mutually recognize each other. Many X-ray co-crystal structures of protein–DNA complexes are now known, so we have a good idea of the structure of the final complex. These co-crystal structures reveal readout mechanisms for many families of DNA-binding proteins, each having distinct structural features and modes of binding (1). But because there are relatively few high-resolution structures of naked DNA, and only a handful of structures of DNA having the precise sequence that is found in a protein–DNA complex (2), we know surprisingly little about the structure of the DNA molecule that a protein must recognize before binding. This question has come to the fore with the growing appreciation of the role that DNA shape plays in binding site recognition and protein binding (3–7).

In this paper, we asked whether pre-existing structural features of a DNA binding site persist in a protein–DNA complex, or whether the structure of the DNA changes substantially upon protein binding. The first case represents shape recognition, and the second induced fit.

To answer this question for the universe of protein–DNA complexes, it will be necessary to have detailed structural information for a very large number of naked DNA molecules, to compare with the structure of DNA in protein–DNA complexes. While high-resolution 3D structural information would of course be ideal, it is unlikely that crystal or NMR structures will be obtained for such a large number of DNA sequences. Here, we describe an experimental approach, much higher in throughput compared to X-ray crystallography and NMR spectroscopy, that provides a nucleotide-resolution map of one important shape feature of DNA, the width of the minor groove. While minor groove shape is only one of the structural features of DNA, it has been demonstrated that a narrow minor groove is widely exploited by DNA-binding proteins because of its negative electrostatic potential (8).

To generate an experimental map of minor groove width we treat a naked DNA duplex with the hydroxyl radical. The hydroxyl radical abstracts a hydrogen atom from a deoxyribose along the DNA backbone, thereby causing a strand break (9). Because the hydroxyl radical cleaves DNA without regard for the identity of the nucleotide, cleavage data are obtained for every nucleotide in a DNA molecule. We have previously shown that the extent of strand cleavage strongly correlates with the width of the minor groove (10).

We constructed a 399 base-pair (bp) DNA molecule that contained 11 protein-binding sites, separated by short spacer sequences. We used capillary electrophoresis to separate and quantify the products of hydroxyl radical cleavage. Our experiment allowed us to compare the minor groove width of naked DNA with the minor groove width of the same DNA sequence in complex with protein. We found that in each of the 11 binding sites, a region of narrow minor groove width that is present in naked DNA persists in the protein–DNA complex, supporting the idea that the shape of the DNA minor groove is an intrinsic recognition element for DNA-binding proteins. In a few of the complexes we found evidence that protein binding changes the shape of the minor groove, suggesting that in these complexes induced fit also contributes to binding site recognition.

## MATERIALS AND METHODS

### Design and synthesis of the DNA construct

The total length of the plasmid insert containing the target DNA sequence was 399 bp, including a restriction site (BamHI and HindIII) at each end. Sequences of the transcription factor binding sites included in the DNA molecule are listed in Supplementary Table S1 in the order in which they are arranged on the plasmid forward strand. The full plasmid insert sequence used in this study was:

GGATCCGGCTGAAGGTACAGACCCTTTAGTCA GTCTAGGATCATATGCCCAAACGGAACCCCAG CTGTGATTTATGGCGTGGTTACATGTAAAAATTT ACATCTTAGACCCACATTTGAAAGGCAAATGG AGTACGTGTTTTTTAAAAAAATGTCCACGGGG GTCCTATAGAACTTTCCCACAGAGTATAGTACAA ACTTTCTTGTATATAACTCACTAATTGAAGGCGC GAATTCGCGGTATGCAAATAAGGGATGCGTCC TCATGTATATACATGAGGAAGCGTGTTAGCTG TCATAAAGTTGTCACGGAGCGCAATTACCTAA TAGGGAAATTTACACGCTAGGGACGCTATTAT CGCTATTAGTATAGCACGATACACGAAAACGC AGGAAGCTT

The sequence of the plasmid insert is listed in Supplementary Table S2 in FASTA format.

The designed DNA sequence was synthesized by Integrated DNA Technologies and inserted into the pIDTSmart plasmid cloning vector, which also contained the *ampR* gene, the pUC origin, a BamHI restriction site immediately 5′ of the insert, and a HindIII restriction site immediately 3′ of the insert. This plasmid was used to transform *Escherichia coli*. Plasmid DNA was prepared from an *E. coli* culture by standard methods (see Supplementary Materials and Methods for details).

The forward (‘P3F’) and reverse (‘P3R’) primer sequences for amplification of the 399 bp DNA molecule, designed using Primer3 (11), were 5′-GGCTGAAGGTACAGACCCTTT-3′ and 5′-CCTGCGTTTTCGTGTATCG-3′, respectively. HPLC-purified Cy5-labeled and unlabeled primers were purchased from Integrated DNA Technologies and were used without further purification. The 399 bp plasmid insert was amplified by PCR and purified by standard methods (see Supplementary Materials and Methods for details).

### Hydroxyl radical cleavage

Hydroxyl radical cleavage reactions (12) were performed in a 96-well plate and automated using a Biomek 3000 Automated Workstation (Beckman Coulter) equipped with a multi-channel pipet tool and a gripper for 96-well plates. Each reaction contained 40 μl (∼5 pmol) of purified, singly fluorescently end-labeled PCR product. For a typical cleavage reaction, 3 μl each of 10 mM sodium ascorbate, 6% H_2_O_2_, and 40 μM iron(II)–EDTA were added to a well containing the DNA solution. The amounts of iron(II)–EDTA and H_2_O_2_ used in the cleavage reaction were optimized to achieve single-hit kinetics and avoid destruction of the fluorophore. Reactions were carried out for 2 min and then stopped by the addition of 10 μl 0.4 M thiourea. DNA was purified using the same magnetic bead cleanup step used for PCR cleanup (see Supplementary Materials and Methods). Following bead cleanup, the DNA sample was dried by vacuum centrifugation (SpeedVac).

### Capillary gel electrophoresis

In a 96-well plate, the dried, hydroxyl radical-cleaved, DNA sample was resuspended in 40 μl sample loading solution (SLS; Beckman Coulter) containing 0.5 μl Genome Size Standard 400 (Beckman Coulter). The solution was mixed thoroughly, and one drop of mineral oil was placed into the well to prevent sample evaporation. In a separate 96-well round-bottom plate, wells were half-filled with running buffer (Beckman Coulter). Sample and buffer plates were loaded onto a CEQ 8000 capillary electrophoresis instrument (Beckman Coulter). The CEQ manifold and capillary array were purged with 0.5–1.2 ml polyacrylamide/urea gel solution (Beckman Coulter) prior to electrophoresis. The sample-containing 96-well plate was heated for 2.5 min at 90°C within the instrument to denature the DNA. Sample injections were performed at 2 kV for 7 s. Electrophoresis was carried out for 1.5 h at a voltage of 2 kV and a capillary temperature of 60°C. Fluorescence data were acquired at a rate of 2 Hz.

### Data processing and peak integration

We wrote a custom MATLAB application to visualize raw electrophoresis data and fit and integrate peaks. The code for this application (which we call RobFinder) is freely available on GitHub, at https://github.com/rnaplus/RobFinder. We also have used other software packages (including ShapeFinder (13) and QuShape (14)) to process capillary electrophoresis data, with similar results.

To process a dataset using RobFinder, the raw fluorescence intensities for the size-standard ladder and hydroxyl radical-treated DNA samples were loaded into the application. Baseline subtraction was performed on each data channel by subtracting the global minimum intensity value from all data points in the trace. Ladder peak assignments were made automatically using the known lengths of the DNA fragments in the Beckman Coulter Genome Size Standard and a simple peak detection routine that uses a sensitivity parameter to find peak maxima. A non-linear least squares method was used to fit the size standard data to a summation of Gaussians that had the form:

\begin{equation*}G\ \left( x \right) = {\rm{\ }}b + \mathop \sum \limits_{i\ = {\rm{\ }}1}^n \left( {{a_i}{\rm{*\ }}{e^{ - 0.5{{\left( {\frac{{x - {c_i}}}{{{w_i}}}} \right)}^2}}}} \right)\end{equation*}

where *b* is the baseline value, *i* is the peak number, *n* is the total number of peaks, *a* is the peak amplitude, *c* is the peak center, and *w* is the peak width. The baseline value was either initialized to zero or to a value chosen manually from within the application. Starting peak parameters for hydroxyl radical cleavage data were estimated from those derived from the size standards. Specifically, peak centers (*c*) were linearly interpolated between consecutive size standard peaks, each peak amplitude (*a*) was set equal to the fluorescence intensity value in the hydroxyl radical-treated channel for each corresponding peak center, and peak widths (*w*) were obtained via linear regression of the size standard peak widths versus data point. Initial peak locations were manually inspected and coarsely adjusted when necessary.

Hydroxyl radical peak intensities were fit by the Gaussian model function using a non-linear least squares method function (*lsqcurvefit*) from the MATLAB Optimization Toolbox. Three passes through the peak optimization routine were employed: (i) to simultaneously optimize parameters *a, c* and *w* in order to obtain a confidence interval for the linear regression of peak width versus data point; (ii) to constrain peak widths to within the confidence interval bounds while optimizing parameters *a* and *c*; (iii) to optimize *a* and *c* while keeping the peak widths *w* fixed. Each pass was performed by fitting peaks within a sliding window of 600 data points (∼40–60 peaks) that was shifted by 300 points in consecutive iterations across the length of the electropherogram. Parameters optimized for the first five and last five peaks within each window were discarded to eliminate fitting bias.

Each final, optimized peak was integrated using a trapezoidal approximation (MATLAB *trapz*) over the entire fitted range. Raw peak areas were normalized by dividing each individual peak area value by the median value within a window of 50 nucleotides. Normalization in this manner sets the median peak area within each 50-bp window to a hydroxyl radical cleavage value of 1.000, while preserving the dynamic range of the cleavage values for individual peaks.

### Assignment of nucleotide sequence to the hydroxyl radical cleavage pattern

To assign the nucleotide identity of each peak in the cleavage pattern, we took advantage of the deuterium kinetic isotope effect on hydroxyl radical cleavage that we have previously reported. We had found that substitution of deuterium for the two hydrogen atoms attached to the 5′-carbon atom of a deoxyribose residue results in a decrease of nearly a factor of two in hydroxyl radical-induced cleavage (9). We prepared a fluorescently-labeled 399-mer DNA sample specifically deuterated at each adenine by performing PCR using [5′,5″-D_2_]dATP in place of natural dATP. To assign the nucleotide sequence to the cleavage pattern, we compared the cleavage patterns of the normal and deuterated DNA samples. Peaks that differed substantially in intensity between the two patterns were labeled as adenine. Other peaks were assigned by interpolation of the known nucleotide sequence between assigned adenines.

### Generation of the experimental ORChID2 pattern for the 399-mer

We previously showed that appropriately averaging the hydroxyl radical cleavage values of the two strands of a DNA duplex provides an experimental map of the variation in minor groove width. We called this pattern ORChID2 (**O**H **R**adical **C**leavage **I**ntensity **D**atabase, **2** strands) (10). To produce the experimental ORChID2 pattern (which we call expORChID2) for the 399-mer DNA molecule, for each position in the sequence, we took the integrated and normalized cleavage value for the nucleotide on one strand and averaged it with the cleavage value for the nucleotide on the other strand that is shifted three nucleotides in the 3′ direction. Because of the geometry of B-form DNA, these two nucleotides are directly across the minor groove from each other.

### Generation of the computed ORChID2 pattern for the 399-mer

We used the Perl scripts that are available for download on the website http://dna.bu.edu/orchid to calculate the ORChID2 pattern (15) for the 399-bp DNA sequence. The computed ORChID2 pattern (which we call compORChID2) for genomic sites can also be derived from our GBshape database at http://rohsdb.usc.edu/GBshape/ (16).

### Loess smoothing of the ORChID2 pattern

We found that minimally smoothing the ORChID2 pattern (both experimental and computed) made for easier comparison with minor groove width patterns from X-ray crystallography and from prediction by DNAshape (17), and with each other. To smooth an expORChID2 or compORChID2 pattern we used the *loess.smooth* function in R, with parameters *span* = 0.015 and *evaluation* = 300, to smooth 300 nucleotides in the ORChID2 pattern that encompass the 11 protein binding sites (Supplementary Figure S1).

### Calculation of minor groove width from X-ray co-crystal structures

We measured the minor groove width of double-stranded DNA in co-crystal structures using CURVES (18). Minor groove width is defined as the minimum distance between phosphodiester backbone atoms minus 5.8 Å, which represents the sum of the phosphate van der Waals radii in opposite strands. To calculate minor groove width as a function of sequence, we averaged the values assigned to a given nucleotide position using CURVES (18) default parameters. This definition allows comparison with the analysis of the identical binding sites in a previous study (8). The PDB IDs of the protein–DNA X-ray co-crystal structures used in this study are listed in Supplementary Table S1.

### Prediction of the minor groove width pattern of naked DNA using DNAshape

To assess the intrinsic shape in unbound DNA, we predicted the minor groove width at each nucleotide position for the 399-bp DNA sequence using our high-throughput method DNAshape (17). The method uses a sliding pentamer window to define a feature vector of minor groove width. The values of the feature vector as a function of its pentamer sequence were derived from all-atom Monte Carlo simulations of naked B-DNA structures of 10–27 bp in length for 2121 different DNA sequences that cover each pentamer on average 44 times (17). These MC simulations followed a previously published protocol (19) based on the AMBER force field using collective and internal degrees of freedom in combination with implicit solvent, explicit sodium counter ions, and associated Jacobians (20). The R/Bioconductor package DNAshapeR (21), used for the prediction of minor groove width for naked DNA, is available at http://www.bioconductor.org/packages/devel/bioc/html/DNAshapeR.html.

### Statistical analysis

To quantitatively assess the similarity of two groups of data points (e.g. the expORChID2 pattern and the pattern of minor grove width from an X-ray co-crystal structure of a protein–DNA complex), we used the Spearman's rank correlation coefficient (Spearman's *ρ*).

To determine the significance of a Spearman's rank correlation between two groups of data points, we applied *t*-test hypothesis testing to the correlation and calculated the corresponding *P-*value. *P* was obtained by regression analysis, based on the rankings of data points for each group. The confidence level *α* was used to determine statistical significance, where *P* ≤ *α* is considered statistically significant. Standard values for *α* are 0.1 (*), 0.05 (**), 0.01 (***) and 0.001 (****). For example, there is a highly significant correlation between expORChID2 values and X-ray-derived minor groove widths for the Ubx-Exd heterodimer DNA binding site, because *P* = 0.00026 falls below a confidence level of 0.001.

## RESULTS

**Figure 1.**
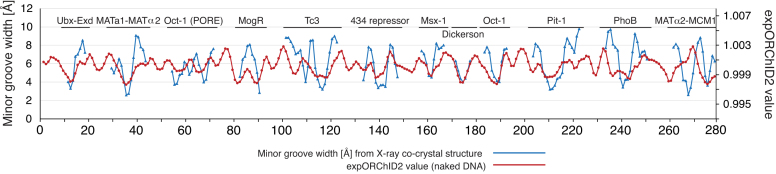
Comparison of the patterns of DNA minor groove width variation in naked DNA and in protein–DNA complexes. Blue, minor groove width measured from X-ray co-crystal structures of protein–DNA complexes; red, the ORChID2 pattern determined experimentally for a 399-bp DNA molecule containing 11 protein–DNA binding sites and the Drew-Dickerson dodecamer sequence (Dickerson).

We began by comparing the experimental ORChID2 pattern for the 399-bp DNA molecule with minor groove widths calculated from X-ray crystal structures of 11 protein–DNA complexes (Figure 1). To facilitate comparison of these two disparate datasets (expORChID2 values for each bp, in arbitrary units, and minor groove widths for X-ray co-crystal structures, in Å), we took advantage of the Drew-Dickerson dodecamer sequence (Dickerson) that we placed near the center of the DNA molecule. We used the minor groove width pattern from the X-ray structure of this naked DNA molecule (10) as a reference to adjust the scale of the *y*-axis of the plot of the expORChID2 dataset, so that, for each nucleotide of the Dickerson dodecamer, the crystallographically-determined minor groove width and the expORChID2 value coincide closely.

We note that this scale adjustment was made only to facilitate initial visual comparison (Figure 1); we base the detailed analysis below on the correspondence of the pattern of minor groove width in the X-ray structure of a protein–DNA complex, with the expORChID2 pattern for that same sequence as naked DNA. We assessed the correspondence of two patterns by calculating the Spearman's *ρ* value, which is a measure of the rank correlation of the values of the two patterns, but which does not depend on the absolute values of expORChID2 or minor groove width.

The first question we asked was, overall, how do the two patterns shown in Figure 1 compare? Visually, we noted that in many sites where the minor groove was narrow in the structure of a protein–DNA complex, there also was a minimum in the expORChID2 pattern for the naked DNA molecule. To more quantitatively investigate this relationship, we calculated the Spearman's *ρ* for each protein binding site and the Drew-Dickerson sequence, comparing the patterns of minor groove width and expORChID2 values, and then averaged the Spearman's *ρ* values over all 12 sites. We found an average Spearman's *ρ* of 0.60 for the overall comparison (171 bp in total), reinforcing the initial impression that there was a notable similarity in the pattern of minor groove widths in a protein–DNA complex and the expORChID2 values for the same sequence as naked DNA. It is clear, though, just by looking at Figure 1, that for a few protein-binding sites the correlation is poor, so we next analyzed each binding site individually (Supplementary Table S3).

### DNA binding sites that have the same shape in the protein–DNA complex as in naked DNA

**Figure 2.**
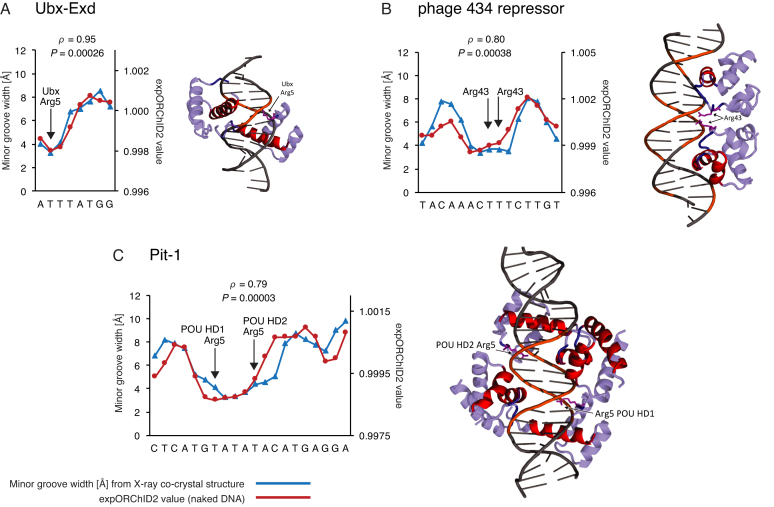
For some protein–DNA complexes, the pattern of minor groove width variation is similar to that of the same sequence as naked DNA. Patterns were quantitatively compared by computing the Spearman's rank correlation coefficient *ρ* and the *P-*value for the similarity. The *y*-axis scale for expORChID2 values differs slightly between plots to facilitate comparison of individual patterns. This does not affect the calculation of the Spearman's rank correlation coefficient (see Materials and Methods). Red filled circles, expORChID2 values; blue filled triangles, minor groove width measured from the protein–DNA complex. Arrows, locations of arginine residues bound to the minor groove in the protein–DNA complex, for reference. (**A**) Ubx-Exd; (**B**) Phage 434 repressor; (**C**) Pit-1.

**Figure 3.**
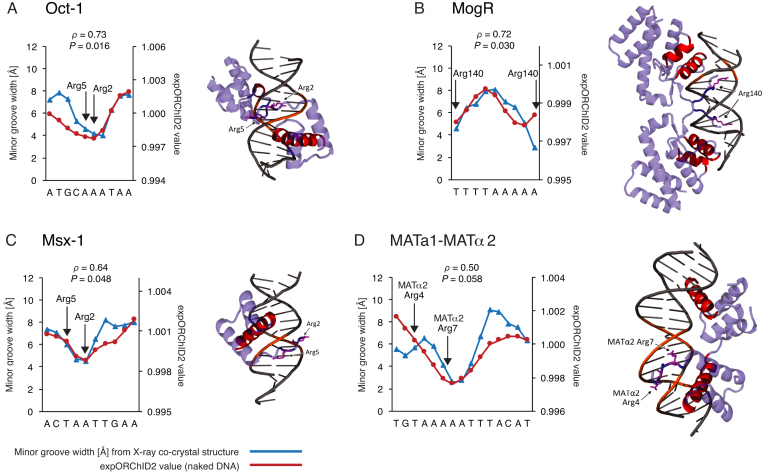
For some protein–DNA complexes, the pattern of minor groove width variation is similar to that of the same sequence as naked DNA. Patterns were quantitatively compared by computing the Spearman's rank correlation coefficient *ρ* and the *P*-value for the similarity. The *y*-axis scale for expORChID2 values differs slightly between plots to facilitate comparison of individual patterns. This does not affect the calculation of the Spearman's rank correlation coefficient (see Materials and Methods). Red filled circles, expORChID2 values; blue filled triangles, minor groove width measured from the protein–DNA complex. Arrows, locations of arginine residues bound to the minor groove in the protein–DNA complex, for reference. (**A**) Oct-1; (**B**) MogR; (**C**) Msx-1; (**D**) MATa1-MATα2.

We first examined binding sites for which the pattern of minor groove width variation in the X-ray co-crystal structure correlated well with the experimental ORChID2 pattern of the naked DNA site. We assessed the similarity of the two patterns by evaluating the Spearman's *ρ* for each site individually (Supplementary Table S3). In addition, we evaluated the significance level of that similarity using a *t*-test (see Materials and Methods for details). Of the 11 binding sites we investigated, we judged seven to have very similar expORChID2 and minor groove width patterns. Spearman's *ρ* values for these binding sites range from 0.50 to 0.95 (Figures 2 and 3). For reference, the Spearman's *ρ* value for the Dickerson sequence (Supplementary Figure S2) was 0.97. The similarity in patterns was highly significant (*P* < 0.001) for all binding sites shown in Figure 2 and significant (*P* < 0.05) for most target sites in Figure 3 (except for panel D; although we note that the similarity between patterns for the MATα2 half-site where arginine residues contact the minor groove was highly significant (Supplementary Table S3)). We conclude that it is likely that, as naked DNA, these 7 DNA binding sites (for the Ubx-Exd heterodimer (22), phage 434 repressor (23), Pit-1 (24), Oct-1 (the octamer site) (25), MogR (26), Msx-1 (27), and the MATα2 half-site (28)) have an intrinsic narrow minor groove region that is recognized by the protein, and that is retained in the protein–DNA complex.

### DNA binding sites that change shape upon protein binding

**Figure 4.**
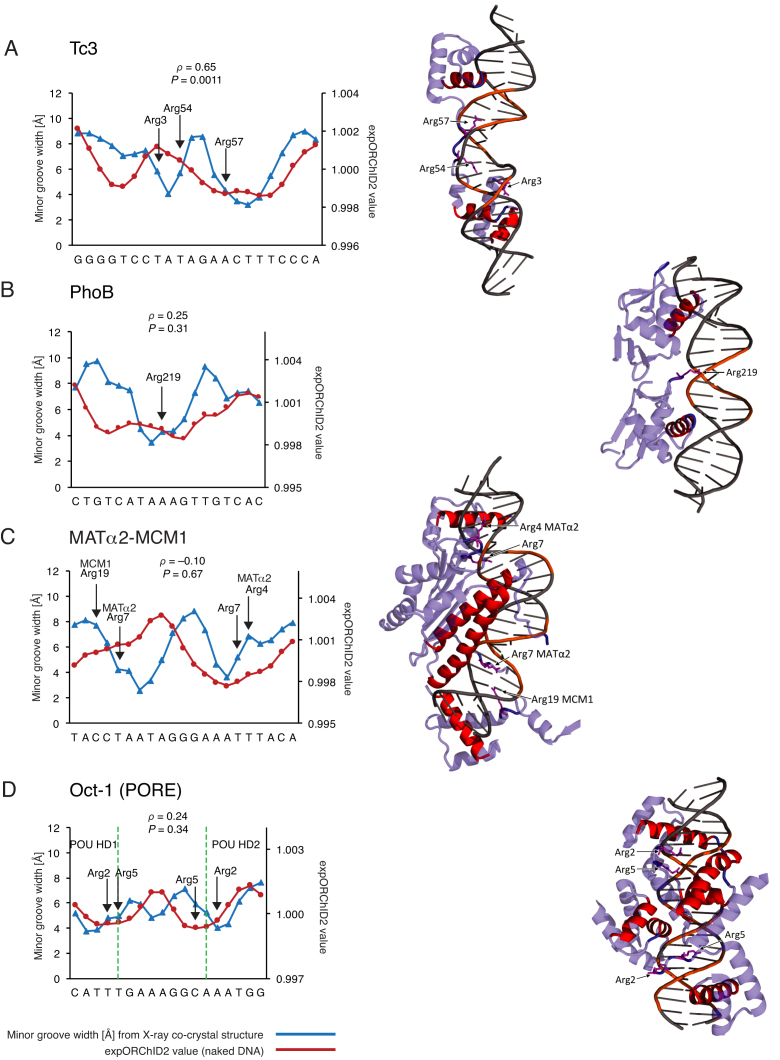
For some protein–DNA complexes, the pattern of minor groove width variation in part of the binding site is similar to that of the same sequence as naked DNA, and in part of the binding site the pattern is different when protein is bound. Patterns were quantitatively compared by computing the Spearman's rank correlation coefficient *ρ* and the *P*-value for the similarity. The *y*-axis scale for expORChID2 values differs slightly between plots to facilitate comparison of individual patterns. This does not affect the calculation of the Spearman's rank correlation coefficient (see Materials and Methods). Red filled circles, expORChID2 values; blue filled triangles, minor groove width measured from the protein–DNA complex. Arrows, locations of arginine residues bound to the minor groove in the protein–DNA complex, for reference. (**A**) Tc3; (**B**) PhoB; (**C**) MATα2-MCM1; (**D**) Oct-1 (PORE). Dashed green lines in (D) demarcate segments of the binding site that interact with the POU-homeodomains (left and right sides) and the POU-specific domains (center) of the Oct-1 (PORE) binding site (see Supplementary Figure S6 for more details).

The other four binding sites we studied show evidence for a combination of shape recognition of a narrow minor groove region that is present in naked DNA, and protein binding-associated structural changes in other segments of the DNA (Figure 4). In the Tc3 transposase target site (29) (Spearman's *ρ* = 0.65; *P* < 0.005), two narrow minor groove regions are seen in both the X-ray structure and in the expORChID2 pattern, but they are spaced differently (Figure 4A). While one of the narrow minor groove regions (the one on the right side of the plot in Figure 4A) is positioned similarly in the complex and in naked DNA, in the protein–DNA complex the two narrow minor groove regions are separated by a half-turn of the DNA helix, while in naked DNA they are separated by a full helical turn.

In the PhoB binding site (30) (Spearman's *ρ* = 0.25), a narrow minor groove region is present in the center of both patterns (Figure 4B). The narrow minor groove region is substantially broader in the experimental ORChID2 pattern (10 bp) than in the X-ray structure (4–5 bp).

We studied two binding sites for heterodimers of the MATα2 protein, the MATa1-MATα2 site (28) (Figure 3D) and the MATα2-MCM1 site (31) (Figure 4C). In both binding sites the half-site to which MATα2 binds has the same minor groove width pattern in naked DNA as it does in the protein–DNA complex (Supplementary Figure S3).

While the Spearman's *ρ* for the complete MATα2-MCM1 site (Figure 4C) is –0.10, when considering only the MATα2 half-site (Supplementary Figure S3), the *ρ* value is 0.86 (Supplementary Table S3). The similarity of the patterns was highly significant (*P* < 0.001) for the MATα2 half-site (right side of the site in Figure 4C). A prominent narrow minor groove region in the MCM1 half-site of the X-ray structure (the left side of the site in Figure 4C) was not present in the expORChID2 pattern, leading to a very low Spearman's rank correlation for the entire site.

The Oct-1 (PORE) binding site (32) showed a poor Spearman's *ρ* (0.24) between the expORChID2 pattern and the minor groove width pattern in the protein–DNA complex (Figure 4D). In the X-ray co-crystal structure, the DNA binding site has three narrow minor groove regions, each separated by a half-turn of the DNA helix. The expORChID2 pattern, in contrast, shows two broad minima separated by nearly a turn of the DNA helix. We noticed, though, that at both the extreme right- and left-hand edges of the binding site (demarcated by dashed green lines in Figure 4D), minima in the two patterns coincide. The center of the site is where the two patterns differ most (see below for discussion of this observation).

### Comparison of experimental and predicted patterns of minor groove width for naked DNA

To allow us to compare two distinct approaches, experimental and computational, for obtaining structural information for naked DNA, we used our DNAshape method (17) to predict the pattern of minor groove width variation for the unbound DNA molecule. We first compared the expORChID2 and DNAshape-predicted minor groove width patterns for 285 bp of the DNA molecule, and found an overall Spearman's *ρ* of 0.25.

We then compared the two patterns in the unbound state for each protein-binding site individually (Supplementary Table S4). We found that for seven of the protein binding sites the patterns were very similar whether determined experimentally or predicted computationally (Supplementary Figure S4A-G). The Spearman's *ρ* values for these comparisons were for Ubx-Exd, 0.71; phage 434 repressor, 0.51; Tc3 transposase, 0.74; PhoB, 0.41; MATa1-MATα2, 0.57; MATα2-MCM1, 0.39; MogR, 0.84 (see Supplementary Table S4 for significance levels).

Two additional cases illustrate the limitation of relying solely on the Spearman's *ρ* value to assess the similarity of two patterns. The experimental and predicted patterns for the Oct-1 and Msx-1 sites appeared similar (Supplementary Figure S4H, I). In each case, the experimental and the predicted pattern was characterized by a single minimum at nearly the same location in the DNA sequence. But because the experimental and predicted patterns differed slightly in the location of the minima, the Spearman's *ρ* values (0.18 and 0.02, respectively) were poor. Given the shift of the minima by a single bp in both cases, despite the apparently poor Spearman's *ρ* values we concluded that the DNAshape and expORChID2 patterns for the Oct-1 and Msx-1 sites agreed. Nine of the 11 binding sites, therefore, had similar DNAshape and expORChID2 minor groove width patterns as naked DNA.

**Figure 5.**
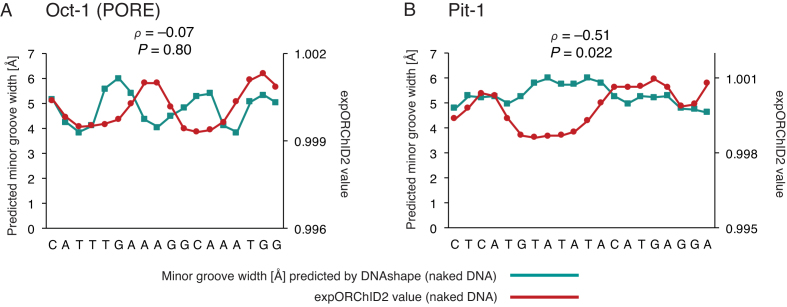
Computational prediction of minor groove width patterns of naked DNA by DNAshape sometimes differs from experimental patterns. Patterns were quantitatively compared by computing the Spearman's rank correlation coefficient *ρ* and the *P*-value for the similarity. The *y*-axis scale for expORChID2 values differs slightly between plots to facilitate comparison of individual patterns. This does not affect the calculation of the Spearman's rank correlation coefficient (see text). Red filled circles, expORChID2 values; teal filled squares, minor groove width predicted by DNAshape for naked DNA. (**A**) Oct-1 (PORE); (**B**) Pit-1.

In two other cases, however, the experimental and predicted patterns differed substantially. For the Oct-1 (PORE) site, the Spearman's *ρ* was –0.07 for comparison of the pattern predicted by DNAshape and the expORChID2 pattern (Figure 5A). We recall that the expORChID2 pattern for Oct-1 (PORE) also correlated poorly with the X-ray co-crystal minor groove width pattern (Figure 4D). The pattern predicted by DNAshape showed three narrow minor groove regions, while the expORChID2 pattern had only two. However, the minor groove width pattern predicted by DNAshape agrees much better with the pattern derived from the X-ray co-crystal structure (Spearman's *ρ* = 0.61) (17). We discuss possible reasons for this discrepancy between expORChID2 and DNAshape below.

In another case, the Pit-1 site (Figure 5B), the Spearman's *ρ* value for comparison of the DNAshape and expORChID2 patterns was –0.51. The difference between the two patterns is that an extended narrow minor groove region in the expORChID2 pattern is not seen in the minor groove width pattern predicted by DNAshape. However, the expORChID2 pattern for the Pit-1 binding site is an excellent match for the minor groove width pattern from the X-ray structure of the Pit-1/DNA complex (Figure 2C), both of which exhibit a similar extended narrow minor groove width region (see below for discussion of this protein target).

### Comparison of experimental and computed ORChID2 patterns

The ORChID approach originated as a computational method to predict the hydroxyl radical cleavage pattern of an input DNA sequence based on a database of experimental cleavage data (15). The ORChID pattern corresponds to one of the DNA strands of a DNA duplex. We later developed ORChID2, which averages the cleavage patterns for the two DNA strands across the minor groove, and showed that there is a strong correlation between the ORChID2 value and minor groove width (10). In this study, we determined the expORChID2 pattern for the 399-bp DNA molecule by experimentally measuring the hydroxyl radical cleavage pattern for each strand, and then averaging the cleavage values across the minor groove. Since we also have developed software to compute the ORChID2 pattern for any input DNA sequence, we compared experimental and computed ORChID2 patterns for 285 bp of the DNA molecule (Supplementary Figure S5), and found a Spearman's *ρ* value of 0.69. This result validates the use of computed ORChID2 patterns to provide an approximate map of the variation of minor groove width in DNA sequences of any length. Indeed, compORChID2 patterns for a large number of genomes (including human) are available in the GBshape database (16).

An interesting point in our comparison of the experimental and computed ORChID2 patterns is that the only major discrepancy appears in the long alternating pyrimidine-purine sequence in the Pit-1 target site, similar to the result we found for comparison of expORChID2 with DNAshape (Figure 5B). We also note that the expORChID2 and compORChID2 patterns for the Oct-1 (PORE) site are similar.

## DISCUSSION

We focused here on the question of whether distinct structural features of DNA in a protein–DNA complex are present in the naked DNA to which the protein binds (2). This is often a difficult question to answer using experimental structural data, because there are very few X-ray crystal structures or NMR structures of naked DNA molecules capturing the sequence of a protein binding site for which a structure of the protein–DNA complex is available (2,33).

It has long been recognized that the structure of DNA in a protein–DNA complex often varies from the canonical B-form. Olson and coworkers published a seminal study in which they analyzed DNA structural parameters from 92 X-ray structures of protein–DNA complexes (34). While their analysis was the first to comprehensively define the deformability of DNA when bound to protein, they were limited by the lack of structural data for the corresponding naked DNA binding sites to which to compare. Jen-Jacobson and coworkers discovered a remarkable correspondence between DNA distortion and the thermodynamic parameters (entropy and enthalpy) associated with protein binding (35). Relatively undistorted DNA in the complex was associated with favorable enthalpy change upon binding, while bound DNA that was highly distorted was associated with highly favorable entropy change. Once again, though, it was not possible to directly assess the extent of DNA distortion associated with protein binding because structures of the naked DNA binding sites were not available. Lawson and coworkers were the first to systematically compare free and bound DNA structures in an effort to reveal protein-induced DNA distortion (2). After performing crystallization screening trials for 50 DNA oligonucleotides having sequences of various protein binding sites, they were able to solve X-ray structures for four sequences, and thereby compare the structures of cognate naked DNA and DNA bound to protein.

We showed previously that a key DNA structural feature, minor groove width, is amenable to experimental determination by analysis of hydroxyl radical cleavage patterns (10). Narrow minor groove width and protein recognition of DNA have a compelling physical connection. A narrow minor groove is associated with enhanced negative electrostatic potential (36), and proteins have been shown to interact with these regions of negative electrostatic potential by inserting one or more positively-charged side chains (arginine (8), lysine (37), or histidine (38)) into the narrow minor groove.

By using high-throughput computational prediction of naked DNA structure, other structural features of DNA (helical parameters, for example) have been found to correlate with protein binding and improve our ability to predict DNA binding specificity (5,7,39). However, experimental methods, other than X-ray crystallography or NMR spectroscopy, that are capable of deriving these structural features are not available. There also is no simple physical picture connecting protein binding to patterns of helical parameters.

We therefore asked whether a region of narrow minor groove width that exists in naked DNA is present in the corresponding protein–DNA complex. The 11 protein binding sites we examined were chosen because they were highlighted in the first study to reveal the generality of shape recognition of narrow minor groove width by proteins (8).

Twelve different proteins recognize these 11 binding sites. Seven of the proteins contain a homeodomain (MATa1, MATα2, Ubx, Exd, Msx-1, Oct-1 and Pit-1). Two of the homeodomain-containing proteins, Oct-1 and Pit-1, are from the POU family, in which a homeodomain (POU-homeodomain) and a helix-turn-helix motif (POU-specific domain) are connected by a linker peptide. The remaining five proteins do not employ a homeodomain for binding: PhoB (winged helix), MogR (helix-turn-helix plus a loop), MCM1 (MADS box), Tc3 transposase (two helix-turn-helix motifs connected by a peptide linker), and bacteriophage 434 repressor (helix-turn-helix).

The Oct-1 (PORE), Pit-1, PhoB, MogR, and bacteriophage 434 repressor sites are bound by a protein homodimer. Two of the DNA sites are bound by a protein heterodimer (Ubx-Exd, MATa1-MATα2), and one is bound by a heterotetramer (MATα2-MCM1). The Oct-1, Tc3 transposase, and Msx-1 sites are each bound by a protein monomer. Images of the protein–DNA complexes are shown in Figures 2–4.

We found that 7 of the 11 DNA sites have very similar minor groove width patterns as naked DNA and in a protein–DNA complex (Figures 2 and 3). The other four sites all had one or more narrow minor groove regions in naked DNA that persist in the complex, and other narrow minor groove regions that were seen only in the complex (Figure 4). In three of these sites, regions of narrow minor groove width were present at the same place in both the complex and naked DNA, while another narrow minor groove region in the protein–DNA complex was not seen in naked DNA (Figure 4A, C, D). In the other site, a broad narrow minor groove width region in naked DNA was much more localized in the protein–DNA complex (Figure 4B).

We therefore suggest that a region of narrow minor groove width that is present in naked DNA is likely to be recognized by a DNA-binding protein and maintained in the protein–DNA complex. In support of that idea, we found that almost every example of a region of narrow minor groove width in a naked DNA binding site coincided with the site of interaction of an arginine residue from the DNA-binding protein in the complex. We marked these arginine interactions with arrows in Figures 2–4. The only exception was the Tc3 site, for which one of the regions of narrow minor groove width in naked DNA (the one on the left of Figure 4A) was not bound by Arg in the complex. However, in the Tc3/DNA complex the minor groove in this region was wider, perhaps the result of protein-induced deformation of DNA structure, and so was less likely to be electrostatically bound by Arg. In cases where the minor groove geometry changed upon protein binding, we suggest that intrinsic shape features of the unbound DNA assisted the protein in locating its binding site (4,40).

We also compared computational prediction of minor groove width in naked DNA using DNAshape (17) with experimental determination by hydroxyl radical cleavage (expORChID2). We found that in 9 of 11 sites the experimental and predicted minor groove width patterns agreed well (Supplementary Figure S4), providing validation for the computational approach of DNAshape (17). A recent study has compared the use of DNAshape-based structural features in quantitative models of DNA binding specificity (41) with the use of equivalent DNA parameters from 1-μs Molecular Dynamics simulations (42) and X-ray co-crystal structures in the Protein Data Bank (43). The highly comparable results when using DNA shape features from unrelated computational and experimental methodologies (41) confirmed the likely generality of our observations based on DNAshape-derived minor groove width.

In two cases the experimental and computationally-predicted minor groove width patterns differed substantially. For the Oct-1 (PORE) site, the pattern of minor groove width predicted by DNAshape matched the pattern in the X-ray structure of the protein–DNA complex, while the experimental ORChID2 pattern differed from both. In the other case, the Pit-1 site, the experimental ORChID2 pattern closely matched the X-ray co-crystal pattern, while the minor groove width pattern of naked DNA predicted by DNAshape differed from both experimental patterns. We suggest that these two cases may be revealing DNA sequences that are capable of adopting multiple conformations that differ little in energy.

Protein binding to the Oct-1 (PORE) site results in a DNA conformation that is similar to that predicted by DNAshape for naked DNA. This pattern is characterized by three regions of narrow minor groove, including a narrow minor groove in the center of the binding site that is not seen in the expORChID2 pattern. The central narrow minor groove occurred where the POU-specific (POU-S) domains of the Oct-1 homodimer contact each other (Figure 4D, Supplementary Figure S6), leading to compression of the minor groove. Because the center of the binding site apparently was readily distorted by protein binding, it is possible that DNAshape predicted for this region a minor groove width pattern that corresponds to an energetically-accessible conformation that was not the same as the conformation detected experimentally by ORChID2 for this sequence when free in solution.

The POU-homeodomains of the Oct-1 homodimer contact the right and left edges of the PORE binding site (Figure 4D, Supplementary Figure S6). In these regions, the minor groove width pattern in the X-ray co-crystal structure, the minor groove width pattern predicted by DNAshape, and the expORChID2 pattern corresponded closely (Supplementary Figure S6). Spearman's *ρ* values were 0.78 and 0.88, with *P* < 0.05 and 0.005, when the expORChID2 pattern was compared with the X-ray or DNAshape pattern, respectively, at the binding site edges (see Supplementary Tables S3 and S4). We conclude that the segments of the binding site recognized by the POU-homeodomain of Oct-1 had an intrinsically narrow minor groove that did not change upon protein binding.

In the case of the Pit-1 target site, an extended narrow minor groove width region was observed in both the expORChID2 pattern and in the X-ray co-crystal structure (Figure 2C), while the minor groove is normal in width in the patterns predicted by DNAshape (Figure 5B) and by compORChID2 (Supplementary Figure S5). At the center of the Pit-1 site there is a stretch of 13 consecutive alternating pyrimidine-purine nucleotides. The sequence T-A-T-A-T-A occurs at the center of this segment. The pyrimidine-purine (Py-Pu) step (particularly T-A), which is considered to be the most flexible bp step, often is called a ‘hinge’ step due to weak stacking interactions (44). The inherent flexibility of the long stretch of Py-Pu steps at the center of the Pit-1 site may offer an energetically accessible conformation to computational prediction that is not seen experimentally for naked DNA.

## CONCLUSIONS

The work we describe here serves to introduce an experimental and computational analysis pipeline for determining an important DNA shape feature, minor groove width, at nucleotide resolution for DNA molecules several hundred bp in length. In particular, we showed that a robotic liquid handling platform can be used to automate the ORChID2 experiment, starting from a PCR reaction mixture and ending with a hydroxyl radical-cleaved DNA sample ready for capillary electrophoretic analysis. Capillary electrophoresis can produce a high-resolution hydroxyl radical cleavage pattern for at least 300 nucleotides in a single experiment, substantially more than by standard gel electrophoresis. To further increase the throughput of this experiment, we currently are developing an analogous workflow that involves the use of high-throughput sequencing to analyze hydroxyl radical cleavage patterns for much larger DNA molecules. Even so, by using the current capillary electrophoresis-based workflow we have more than doubled the number of DNA sequences for which experimental structural data at nucleotide resolution are available both for a naked DNA molecule and for that DNA molecule bound to protein (2).

This new experimental approach for mapping minor groove geometry in solution on a large scale enabled the deciphering of different mechanisms for DNA binding on a protein family-specific basis. Here, we were able to distinguish between proteins that recognize the intrinsic DNA shape of their binding site (shape readout) and other proteins that seem to read DNA deformability and conformational flexibility (induced fit). These insights will reveal readout mechanisms when experimental three-dimensional structures of naked DNA targets are unavailable. This capability will be important in understanding recognition of the variety of DNA binding sites that a given transcription factor binds to in a genome. Often only a single X-ray co-crystal structure per protein is available, in which the protein is bound to one particular DNA sequence. In reality, however, proteins bind with varying affinity to many related DNA sequences (7,45,46). The methods we introduce here will allow us to probe binding mechanisms for an unrestricted number of target DNA sequences.

## AVAILABILITY

The code for the RobFinder application is freely available on GitHub, at https://github.com/rnaplus/RobFinder.

## Supplementary Material

Supplementary Data

## References

[1] Garvie C.W., Wolberger C. Recognition of specific DNA sequences. Mol. Cell. 2001; 8:937–946. 11741530 10.1016/s1097-2765(01)00392-6

[2] Locasale J.W., Napoli A.A., Chen S., Berman H.M., Lawson C.L. Signatures of protein–DNA recognition in free DNA binding sites. J. Mol. Biol. 2009; 386:1054–1065. 19244617 10.1016/j.jmb.2009.01.007 PMC2753591

[3] Slattery M.G., Zhou T., Yang L., Dantas Machado A.C., Gordân R., Rohs R. Absence of a simple code: how transcription factors read the genome. Trends Biochem. Sci. 2014; 39:381–399. 25129887 10.1016/j.tibs.2014.07.002 PMC4149858

[4] Zentner G.E., Kasinathan S., Xin B., Rohs R., Henikoff S. ChEC-seq kinetics discriminates transcription factor binding sites by DNA sequence and shape in vivo. Nat. Commun. 2015; 6:8733. 26490019 10.1038/ncomms9733 PMC4618392

[5] Mathelier A., Xin B., Chiu T.P., Yang L., Rohs R., Wasserman W.W. DNA shape features improve transcription factor binding site predictions in vivo. Cell Syst. 2016; 3:278–286. 27546793 10.1016/j.cels.2016.07.001 PMC5042832

[6] Stormo G.D., Roy B. DNA structure helps predict protein binding. Cell Syst. 2016; 3:216–218. 27684185 10.1016/j.cels.2016.09.004

[7] Yang L., Orenstein Y., Jolma A., Yin Y., Taipale J., Shamir R., Rohs R. Transcription factor family-specific DNA shape readout revealed by quantitative specificity models. Mol. Syst. Biol. 2017; 13:910. 28167566 10.15252/msb.20167238 PMC5327724

[8] Rohs R., West S.M., Sosinsky A., Liu P., Mann R.S., Honig B. The role of DNA shape in protein–DNA recognition. Nature. 2009; 461:1248–1253. 19865164 10.1038/nature08473 PMC2793086

[9] Balasubramanian B., Pogozelski W.K., Tullius T.D. DNA strand breaking by the hydroxyl radical is governed by the accessible surface areas of the hydrogen atoms of the DNA backbone. Proc. Natl. Acad. Sci. U.S.A. 1998; 95:9738–9743. 9707545 10.1073/pnas.95.17.9738 PMC21406

[10] Bishop E.P., Rohs R., Parker S.C.J., West S.M., Liu P., Mann R.S., Honig B., Tullius T.D. A map of minor groove shape and electrostatic potential from hydroxyl radical cleavage patterns of DNA. ACS Chem. Biol. 2011; 6:1314–1320. 21967305 10.1021/cb200155t PMC3241897

[11] Untergasser A., Cutcutache I., Koressaar T., Ye J., Faircloth B.C., Remm M., Rozen S.G. Primer3—new capabilities and interfaces. Nucleic Acids Res. 2012; 40:e115. 22730293 10.1093/nar/gks596 PMC3424584

[12] Price M.A., Tullius T.D. Using hydroxyl radical to probe DNA structure. Methods Enzymol. 1992; 212:194–219. 1325598 10.1016/0076-6879(92)12013-g

[13] Vasa S.M., Guex N., Wilkinson K.A., Weeks K.M., Giddings M.C. ShapeFinder: a software system for high-throughput quantitative analysis of nucleic acid reactivity information resolved by capillary electrophoresis. RNA. 2008; 14:1979–1990. 18772246 10.1261/rna.1166808 PMC2553743

[14] Karabiber F., McGinnis J.L., Favorov O.V., Weeks K.M. QuShape: Rapid, accurate, and best-practices quantification of nucleic acid probing information, resolved by capillary electrophoresis. RNA. 2013; 19:63–73. 23188808 10.1261/rna.036327.112 PMC3527727

[15] Greenbaum J.A., Pang B., Tullius T.D. Construction of a genome-scale structural map at single-nucleotide resolution. Genome Res. 2007; 17:947–953. 17568010 10.1101/gr.6073107 PMC1891353

[16] Chiu T.P., Yang L., Zhou T., Main B.J., Parker S.C.J., Nuzhdin S.V., Tullius T.D., Rohs R. GBshape: a genome browser database for DNA shape annotations. Nucleic Acids Res. 2015; 43:D103–D109. 25326329 10.1093/nar/gku977 PMC4384032

[17] Zhou T., Yang L., Lu Y., Dror I., Dantas Machado A.C., Ghane T., Di Felice R., Rohs R. DNAshape: a method for the high-throughput prediction of DNA structural features on a genomic scale. Nucleic Acids Res. 2013; 41:W56–W62. 23703209 10.1093/nar/gkt437 PMC3692085

[18] Lavery R., Sklenar H. Defining the structure of irregular nucleic acids: conventions and principles. J. Biomol. Struct. Dyn. 1989; 6:655–667. 2619933 10.1080/07391102.1989.10507728

[19] Zhang X., Dantas Machado A.C., Ding Y., Chen Y., Lu Y., Duan Y., Tham K.W., Chen L., Rohs R., Qin P.Z. Conformations of p53 response elements in solution deduced using site-directed spin labeling and Monte Carlo sampling. Nucleic Acids Res. 2014; 42:2789–2797. 24293651 10.1093/nar/gkt1219 PMC3936745

[20] Sklenar H., Wüstner D., Rohs R. Using internal and collective variables in Monte Carlo simulations of nucleic acid structures: chain breakage/closure algorithm and associated Jacobians. J. Comput. Chem. 2006; 27:309–315. 16355439 10.1002/jcc.20345

[21] Chiu T.P., Comoglio F., Zhou T., Yang L., Paro R., Rohs R. DNAshapeR: an R/Bioconductor package for DNA shape prediction and feature encoding. Bioinformatics. 2016; 32:1211–1213. 26668005 10.1093/bioinformatics/btv735 PMC4824130

[22] Passner J.M., Ryoo H.D., Shen L., Mann R.S., Aggarwal A.K. Structure of a DNA-bound Ultrabithorax-Extradenticle homeodomain complex. Nature. 1999; 397:714–719. 10067897 10.1038/17833

[23] Aggarwal A.K., Rodgers D.W., Drottar M., Ptashne M., Harrison S.C. Recognition of a DNA operator by the repressor of phage 434: a view at high resolution. Science. 1988; 242:899–907. 3187531 10.1126/science.3187531

[24] Jacobson E.M., Li P., Leon-del-Rio A., Rosenfeld M.G., Aggarwal A.K. Structure of Pit-1 POU domain bound to DNA as a dimer: unexpected arrangement and flexibility. Genes Dev. 1997; 11:198–212. 9009203 10.1101/gad.11.2.198

[25] Klemm J.D., Rould M.A., Aurora R., Herr W., Pabo C.O. Crystal structure of the Oct-1 POU domain bound to an octamer site: DNA recognition with tethered DNA-binding modules. Cell. 1994; 77:21–32. 8156594 10.1016/0092-8674(94)90231-3

[26] Shen A., Higgins D.E., Panne D. Recognition of AT-rich DNA binding sites by the MogR repressor. Structure. 2009; 17:769–777. 19446532 10.1016/j.str.2009.02.018 PMC2712671

[27] Hovde S., Abate-Shen C., Geiger J.H. Crystal structure of the Msx-1 homeodomain/DNA complex. Biochemistry. 2001; 40:12013–12021. 11580277 10.1021/bi0108148

[28] Li T., Jin Y., Vershon A.K., Wolberger C. Crystal structure of the MATa1/MATα2 homeodomain heterodimer in complex with DNA containing an A-tract. Nucleic Acids Res. 1998; 26:5707–5718. 9838003 10.1093/nar/26.24.5707 PMC148023

[29] Watkins S., van Pouderoyen G., Sixma T.K. Structural analysis of the bipartite DNA-binding domain of Tc3 transposase bound to transposon DNA. Nucleic Acids Res. 2004; 32:4306–4312. 15304566 10.1093/nar/gkh770 PMC514390

[30] Blanco A.G., Solà M., Gomis-Rüth F.X., Coll M. Tandem DNA recognition by PhoB, a two-component signal transduction transcriptional activator. Structure. 2002; 10:701–713. 12015152 10.1016/s0969-2126(02)00761-x

[31] Tan S., Richmond T.J. Crystal structure of the yeast MATα2/MCM1/DNA ternary complex. Nature. 1998; 391:660–666. 9490409 10.1038/35563

[32] Reményi A., Tomilin A., Pohl E., Lins K., Philippsen A., Reinbold R., Schöler H.R., Wilmanns M. Differential dimer activities of the transcription factor Oct-1 by DNA-induced interface swapping. Mol. Cell. 2001; 8:569–580. 11583619 10.1016/s1097-2765(01)00336-7

[33] Rohs R., West S.M., Liu P., Honig B. Nuance in the double-helix and its role in protein–DNA recognition. Curr. Opin. Struct. Biol. 2009; 19:171–177. 19362815 10.1016/j.sbi.2009.03.002 PMC2701566

[34] Olson W., Gorin A., Lu X., Hock L., Zhurkin V. DNA sequence-dependent deformability deduced from protein–DNA crystal complexes. Proc. Natl. Acad. Sci. U.S.A. 1998; 95:11163–11168. 9736707 10.1073/pnas.95.19.11163 PMC21613

[35] Jen-Jacobson L., Engler L.E., Jacobson L.A. Structural and thermodynamic strategies for site-specific DNA binding proteins. Structure. 2000; 8:1015–1023. 11080623 10.1016/s0969-2126(00)00501-3

[36] Chiu T.P., Rao S., Mann R.S., Honig B., Rohs R. Genome-wide prediction of minor-groove electrostatic potential enables biophysical modeling of protein–DNA binding. Nucleic Acids Res. 2017; 45:12565–12576. 29040720 10.1093/nar/gkx915 PMC5716191

[37] Deng Z., Wang Q., Liu Z., Zhang M., Dantas Machado A.C., Chiu T.P., Feng C., Zhang Q., Yu L., Qi L. Mechanistic insights into metal ion activation and operator recognition by the ferric uptake regulator. Nat.Commun. 2015; 6:7642. 26134419 10.1038/ncomms8642 PMC4506495

[38] Chang Y.P., Xu M., Dantas Machado A.C., Yu X.J., Rohs R., Chen X.S. Mechanism of origin DNA recognition and assembly of an initiator-helicase complex by SV40 large tumor antigen. Cell Rep. 2013; 3:1117–1127. 23545501 10.1016/j.celrep.2013.03.002 PMC3748285

[39] Zhou T., Shen N., Yang L., Abe N., Horton J., Mann R.S., Bussemaker H.J., Gordân R., Rohs R. Quantitative modeling of transcription factor binding specificities using DNA shape. Proc. Natl. Acad. Sci. U.S.A. 2015; 112:4654–4659. 25775564 10.1073/pnas.1422023112 PMC4403198

[40] Dror I., Rohs R., Mandel-Gutfreund Y. How motif environment influences transcription factor search dynamics: Finding a needle in a haystack. Bioessays. 2016; 38:605–612. 27192961 10.1002/bies.201600005 PMC5023137

[41] Li J., Sagendorf J.M., Chiu T.P., Pasi M., Perez A., Rohs R. Expanding the repertoire of DNA shape features for genome-scale studies of transcription factor binding. Nucleic Acids Res. 2017; 45:12877–12887. 29165643 10.1093/nar/gkx1145 PMC5728407

[42] Pasi M., Maddocks J.H., Beveridge D., Bishop T.C., Case D.A., Cheatham T., Dans P.D., Jayaram B., Lankaš F., Laughton C. μABC: a systematic microsecond molecular dynamics study of tetranucleotide sequence effects in B-DNA. Nucleic Acids Res. 2014; 42:12272–12283. 25260586 10.1093/nar/gku855 PMC4231739

[43] Berman H.M., Westbrook J., Feng Z., Gilliland G., Bhat T.N., Weissig H., Shindyalov I.N., Bourne P.E. The Protein Data Bank. Nucleic Acids Res. 2000; 28:235–242. 10592235 10.1093/nar/28.1.235 PMC102472

[44] Crothers D.M., Shakked Z. Neidle S DNA bending by adenine-thymine tracts. Oxford Handbook of Nucleic Acid Structure. 1999; Oxford Oxford University Press 455–470.

[45] Slattery M.G., Riley T., Liu P., Abe N., Gomez-Alcala P., Dror I., Zhou T., Rohs R., Honig B., Bussemaker H.J. Cofactor binding evokes latent differences in DNA binding specificity between Hox proteins. Cell. 2011; 147:1270–1282. 22153072 10.1016/j.cell.2011.10.053 PMC3319069

[46] Abe N., Dror I., Yang L., Slattery M.G., Zhou T., Bussemaker H.J., Rohs R., Mann R.S. Deconvolving the recognition of DNA shape from sequence. Cell. 2015; 161:307–318. 25843630 10.1016/j.cell.2015.02.008 PMC4422406

