# Supplementary material for: Experimental maps of DNA structure at nucleotide resolution distinguish intrinsic from protein-induced DNA deformations
Source: Nucleic Acids Res. 2018 Jan 30;46(5):2636–47. doi: 10.1093/nar/gky033 (PMC5946862; doi:10.1093/nar/gky033)
Supplement: Supplementary Data [file gky033_supp.pdf]

SUPPLEMENTARY DATA

# Experimental maps of DNA structure at nucleotide resolution distinguish intrinsic from protein-induced DNA deformations

Robert N. Azad[1], Dana Zafiropoulos[1], Douglas Ober[1], Yining Jiang[1], Tsu-Pei Chiu[2], Jared M. Sagendorf[2], Remo Rohs[2,*], and Thomas D. Tullius[1,3,*]

[1]Department of Chemistry, Boston University, Boston, MA 02215, USA

[2]Computational Biology and Bioinformatics Program, Departments of Biological Sciences, Chemistry, Physics & Astronomy, and Computer Science, University of Southern California, Los Angeles, CA 90089, USA

[3]Program in Bioinformatics, Boston University, Boston, MA 02215, USA

*To whom correspondence should be addressed. Tel: +1 617 353 2482; Fax: +1 617 353 6466; Email: tullius@bu.edu (T.D.T); or Tel: +1 213 740 0552; Fax: +1 821 4257; Email: rohs@usc.edu (R.R.)

## SUPPLEMENTARY MATERIALS AND METHODS

### Plasmid preparation

The plasmid DNA molecule obtained from Integrated DNA Technologies was resuspended in 80 µL of 10 mM TE buffer (10 mM Tris, pH 7.5, 1 mM EDTA) to a concentration of ~50 ng/µL. The stock solution was diluted 500-fold in nuclease-free water, and 2 µL of this solution was added to a vial of pre-thawed OneShot Top10 Chemically Competent *E. coli* cells (Invitrogen). Bacterial transformation was carried out according to the manufacturer's recommended protocol. Briefly, the plasmid/cell solution was incubated on ice for 30 min, and then heat-shocked by incubating at 42°C for 30 s. Samples were placed immediately on ice. To each vial was added 250 µL pre-warmed SOC medium (included in the kit). After incubating the cells at 37°C for 1 hr with continuous shaking, 5 and 15 µL of the culture were spread onto pre-warmed LB agar

plates containing 100 μg/mL ampicillin sodium salt (Sigma Aldrich). Plates were incubated overnight at 37°C. Individual colonies were selected from a plate and used to separately inoculate 5–8 mL starter cultures containing LB broth and 100 μg/mL ampicillin. Cultures were grown overnight at 37°C with continuous shaking. Plasmid DNA was isolated from the cell culture using the QIAprep Miniprep kit (Qiagen) following the manufacturer's recommended protocol. Plasmid DNA was eluted from the spin column with 50 μL Buffer EB (10 mM Tris·Cl, pH 8.5), with typical recoveries of ~150 ng/μL DNA.

**PCR amplification and purification of 5' end-labeled DNA**

PCR amplification of the insert sequence from whole plasmid DNA was performed in a 96-well plate in a reaction volume of 40 μL per well. The PCR reaction mixture was prepared in a single 1.5 mL microcentrifuge tube, on ice, in a total volume of 320 μL, and then aliquoted into 8 wells of a 96-well plate. Each reaction contained 1X ThermoPol buffer (New England Biolabs), 10% DMSO (vol/vol), 3 mM $MgSO_4$, 200 μM dNTP mix, 0.5 μM P3F and P3R primers, 200 ng DNA template, and 0.3-0.6 U Vent Polymerase (New England Biolabs). PCR thermal-cycling was performed using an initial denaturation step at 90°C for 3 min 45 s, followed by 34 cycles of annealing at 52°C for 40 s and extension at 72°C for 30 s. A final extension step was carried out at 72°C for 5 min before cooling to 4°C for storage. To synthesize a fluorescently labeled PCR product, one of the two primers in the reaction mixture contained Cy5 dye covalently attached at the 5' end, while the other primer was unlabeled.

Post-PCR reaction cleanup was performed using a Biomek 3000 Automated Workstation (Beckman Coulter) equipped with a multi-channel pipet tool and a gripper for 96-well plates. Purification of the PCR product was fully automated and achieved using Agencourt AMPure XP magnetic beads (Beckman Coulter). The Biomek 3000 was programmed to follow the Agencourt-recommended protocol, which consisted of adding 72 μL of resuspended bead solution directly to the PCR mixture, mixing 10 times with a pipet tip, and incubating at room temperature for 5 min. The sample plate was then moved onto a magnet designed to accommodate 96-well plates and allowed to sit for 3 min while the beads were pulled to the inner walls of the wells. The supernatant was discarded and the beads were rinsed 2–3 times with freshly prepared 70% ethanol. After air-drying for 10 min, the plate was transferred off the magnet and the beads were resuspended in 40 μL TE buffer (pH 8.0) to elute the DNA. After 3 min incubation, the plate was transferred back to the magnet, the beads were separated, and the supernatant containing the purified DNA was transferred to a fresh 96-well plate. Absorbance measurements at 260 nm were acquired using a Nanodrop system to quantify the total amount of DNA recovered, typically 20–30 ng/μL.

SUPPLEMENTARY TABLES

**Supplementary Table S1.** Sequences of DNA binding sites in protein–DNA co-crystal structures.

| Protein | Binding Site Sequence | PDB ID |
|---|---|---|
| Ubx-Exd | GTGATTTATGGCG | 1B8I |
| MATa1-MATα2 | CATGTAAAAATTTACATCT | 1AKH |
| Oct-1 (PORE site) | CACATTTGAAAGGCAAATGGAG | 1HF0 |
| MogR | TTTTTTAAAAAAAT | 3FDQ |
| Tc3 transposase | GGGGGTCCTATAGAACTTTCCCACA | 1U78 |
| Phage 434 repressor | AGTACAAACTTTCTTGTAT | 2OR1 |
| Msx-1 | CACTAATTGAAGG | 1IG7 |
| Oct-1 (octamer site) | GTATGCAAATAAGG | 1OCT |
| Pit-1 | TCCTCATGTATATACATGAGGAAG | 1AU7 |
| PhoB | AGCTGTCATAAAGTTGTCACGG | 1GXP |
| MATα2-MCM1 | ATTACCTAATAGGGAAATTTACACG | 1MNM |

**Supplementary Table S2.** Partial sequence of plasmid insert containing protein-binding sites, in FASTA format. 300 nucleotides of the insert are included. Numbering corresponds to plots in Figure 1 and Supplementary Figures S1 and S5.

```
>seq_insert_300_nt
CCCAGCTGTGATTTATGGCGTGGTTACATGTAAAAATTTACATCTTAGACCCACATTTGAAAGGCAAATGGAGTACG
TGTTTTTTAAAAAAATGTCCACGGGGGTCCTATAGAACTTTCCCACAGAGTATAGTACAAACTTTCTTGTATATAAC
TCACTAATTGAAGGCGCGAATTCGCGGTATGCAAATAAGGGATGCGTCCTCATGTATATACATGAGGAAGCGTGTTA
GCTGTCATAAAGTTGTCACGGAGCGCAATTACCTAATAGGGAAATTTACACGCTAGGGACGCTATTATC
```

**Supplementary Table S3.** Statistical comparison between expORChID2 and X-ray structures of protein-DNA complexes

| DNA binding site | Spearman's $\rho$ | $P$-value |
|---|---|---|
| Ubx-Exd | 0.95 | 0.00026 |
| phage 434 repressor | 0.80 | 0.00038 |
| Pit-1 | 0.79 | 0.00003 |
| Oct-1 | 0.73 | 0.01580 |
| MogR | 0.72 | 0.02982 |
| Msx-1 | 0.64 | 0.04791 |
| MATa1-MATα2 | 0.50 | 0.05770 |
| MATa1-MATα2 (MATα2 half-site only) | 0.90 | 0.00016 |
| Tc3 | 0.65 | 0.00112 |
| PhoB | 0.25 | 0.31141 |
| MATα2/MCM1 | –0.10 | 0.67447 |
| MATα2/MCM1 (MATα2 half-site only) | 0.86 | 0.00061 |
| Oct-1 (PORE) | 0.24 | 0.34519 |
| Oct-1 (PORE) (w/o POU-S domain) | 0.78 | 0.01252 |

**Supplementary Table S4.** Statistical comparison between expORChID2 and DNAshape

| **DNA binding site** | **Spearman's *ρ*** | ***P*-value** |
|---:|---:|---:|
| Ubx-Exd | 0.71 | 0.04653 |
| phage 434 repressor | 0.51 | 0.05341 |
| Tc3 | 0.74 | 0.00009 |
| PhoB | 0.41 | 0.09203 |
| MATa1-MATα2 | 0.57 | 0.02682 |
| MATα2/MCM1 | 0.39 | 0.08412 |
| MogR | 0.84 | 0.00468 |
| Oct-1 | 0.18 | 0.62719 |
| Msx-1 | 0.02 | 0.96024 |
| Oct-1 (PORE) | –0.07 | 0.79665 |
| Oct-1 (PORE) (w/o POU-S domain) | 0.88 | 0.00193 |
| Pit-1 | –0.51 | 0.02159 |

SUPPLEMENTARY FIGURES

**Supplementary Figure S1. Loess smoothing of the experimental ORChID2 pattern.** Grey, raw experimental ORChID2 data; black, ORChID2 data smoothed by the *loess.smooth* function in R. 300 data points (nucleotides) were smoothed, using parameters *span*=0.015 and *evaluation*=300.

**Supplementary Figure S2. Comparison of the experimental ORChID2 pattern with the minor groove width pattern determined by X-ray crystallography for the Drew-Dickerson dodecamer sequence.** The experimental ORChID2 pattern (red) was taken from the dataset obtained for the 399-bp DNA molecule (see Fig. 1). The minor groove width pattern (blue) was determined by averaging and symmetrizing minor groove width measurements made on eight X-ray crystal structures of the Drew-Dickerson dodecamer. The Spearman's rank correlation coefficient $\rho$ for comparison of these two patterns was 0.97.

**Supplementary Figure S3. The ORChID2 patterns, and the minor groove width patterns from X-ray co-crystal structures, of two examples of the MATα2 half-site were all very similar to each other.** Red lines, ORChID2 values for the MATα2 half-sites of the MATα2-MCM1 site (filled circles) and the MATa1-MATα2 site (filled triangles). Blue lines, minor groove widths of the MATα2 half-sites measured from X-ray co-crystal structures of the MATα2-MCM1 complex (filled circles) and the MATa1-MATα2 complex (filled triangles). The experimental ORChID2 patterns were taken from the dataset obtained for the 399-bp DNA molecule (see Figure 1). The sequences of the MATα2 half-sites are identical except for the two nucleotides at the 5' end: GG, for the MATα2-MCM1 site; AA, for the MATa1-MATα2 site.

**Supplementary Figure S4. Computational predictions of minor groove width patterns of naked DNA by DNAshape often were highly similar to experimental ORChID2 patterns.** Patterns were quantitatively compared by computing the Spearman's rank correlation coefficient $\rho$ and the *P*-value for the correlation. The *y*-axis scale for expORChID2 values differed slightly between plots to facilitate comparison of individual patterns. This did not affect the calculation of the Spearman's rank correlation coefficient (see Materials and Methods). Red filled circles, expORChID2 values; teal filled squares, minor groove width predicted by DNAshape for naked DNA. **(A)** Ubx-Exd; **(B)** Phage 434 repressor; **(C)** Tc3; **(D)** PhoB; **(E)** MATa1-MATα2; **(F)** MATα2-MCM1; **(G)** MogR; **(H)** Oct-1; **(I)** Msx-1.

**Supplementary Figure S5. Comparison of experimental and computed ORChID2 patterns.** Grey, computed ORChID2 data; black, experimental ORChID2 data. Both patterns were smoothed by the *loess.smooth* function in R. 300 data points (nucleotides) were smoothed, using parameters *span*=0.015 and *evaluation*=300.

**Supplementary Figure S6. The minor groove width patterns of the left and right segments of the Oct-1 (PORE) site did not change upon protein binding.** Red filled circles, expORChID2 values; blue filled triangles, minor groove width measured from the protein–DNA complex; teal filled squares, minor groove width predicted by DNAshape for naked DNA. Arrows, locations of arginine residues bound to the minor groove in the protein–DNA complex,

for reference. Grey box, segment of binding site where the POU-S domains of Oct-1 interact with DNA.

Fig. S1

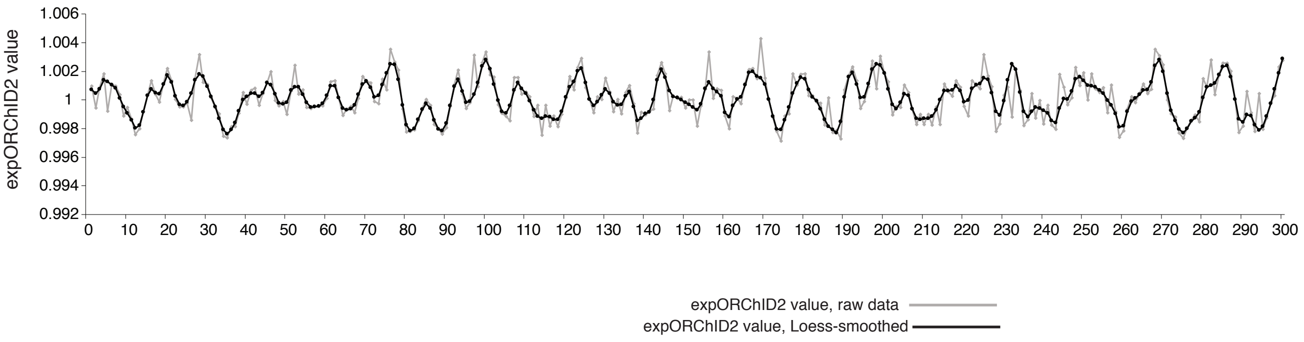
1.006
1.004
1.002
1
0.998
0.996
0.994
0.992
expORChID2 value
0
10
20
30
40
50
60
70
80
90
100
110
120
130
140
150
160
170
180
190
200
210
220
230
240
250
260
270
280
290
300
expORChID2 value, raw data
expORChID2 value, Loess-smoothed

Fig. S2

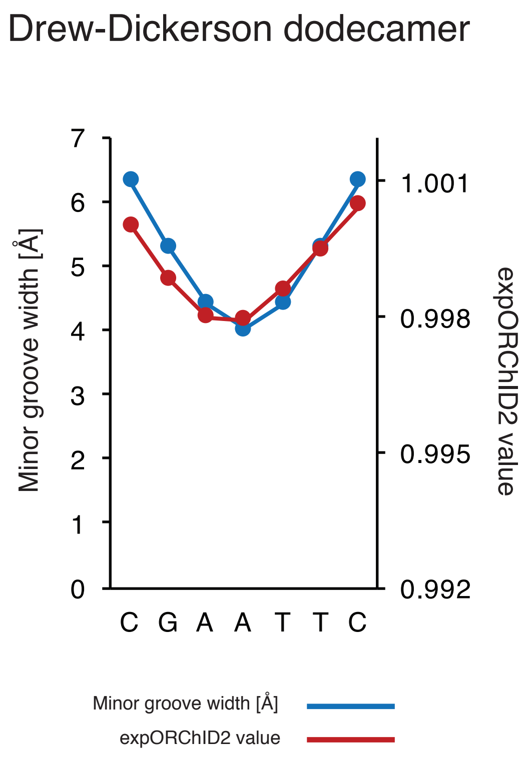

Drew-Dickerson dodecamer
Minor groove width [Å]
expORChID2 value
0
1
2
3
4
5
6
7
0.992
0.995
0.998
1.001
C G A A T T C
Minor groove width [Å]
expORChID2 value

# Fig. S3

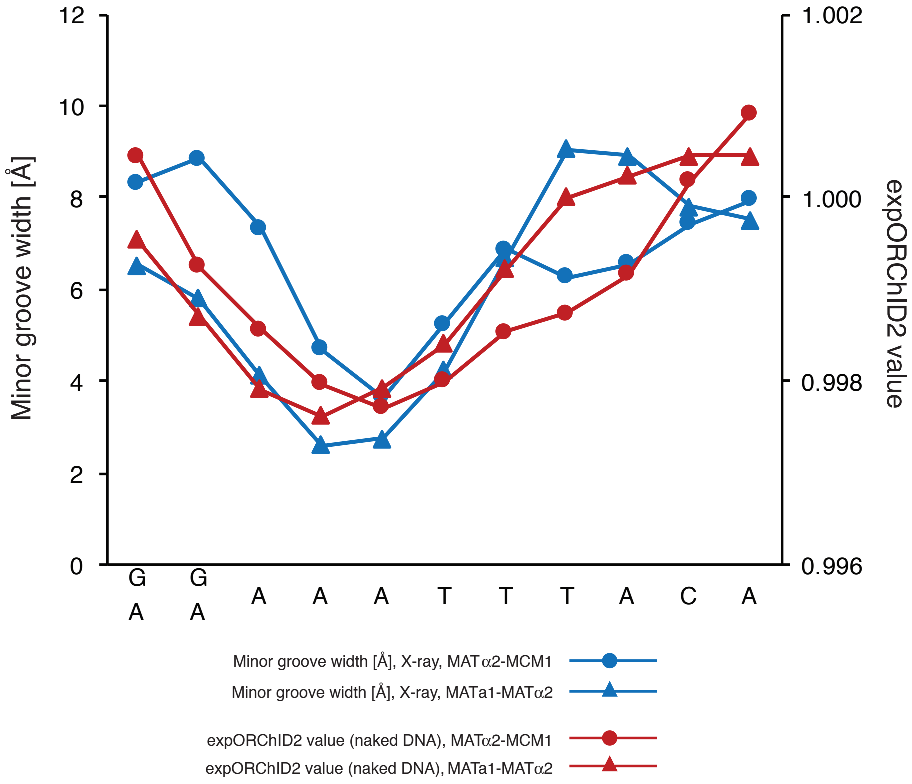

Minor groove width [Å]
expORChID2 value
12
10
8
6
4
2
0
1.002
1.000
0.998
0.996
G
A
G
A
A
A
A
T
T
T
A
C
A
Minor groove width [Å], X-ray, MATα2-MCM1
Minor groove width [Å], X-ray, MATa1-MATα2
expORChID2 value (naked DNA), MATα2-MCM1
expORChID2 value (naked DNA), MATa1-MATα2

# Fig. S4

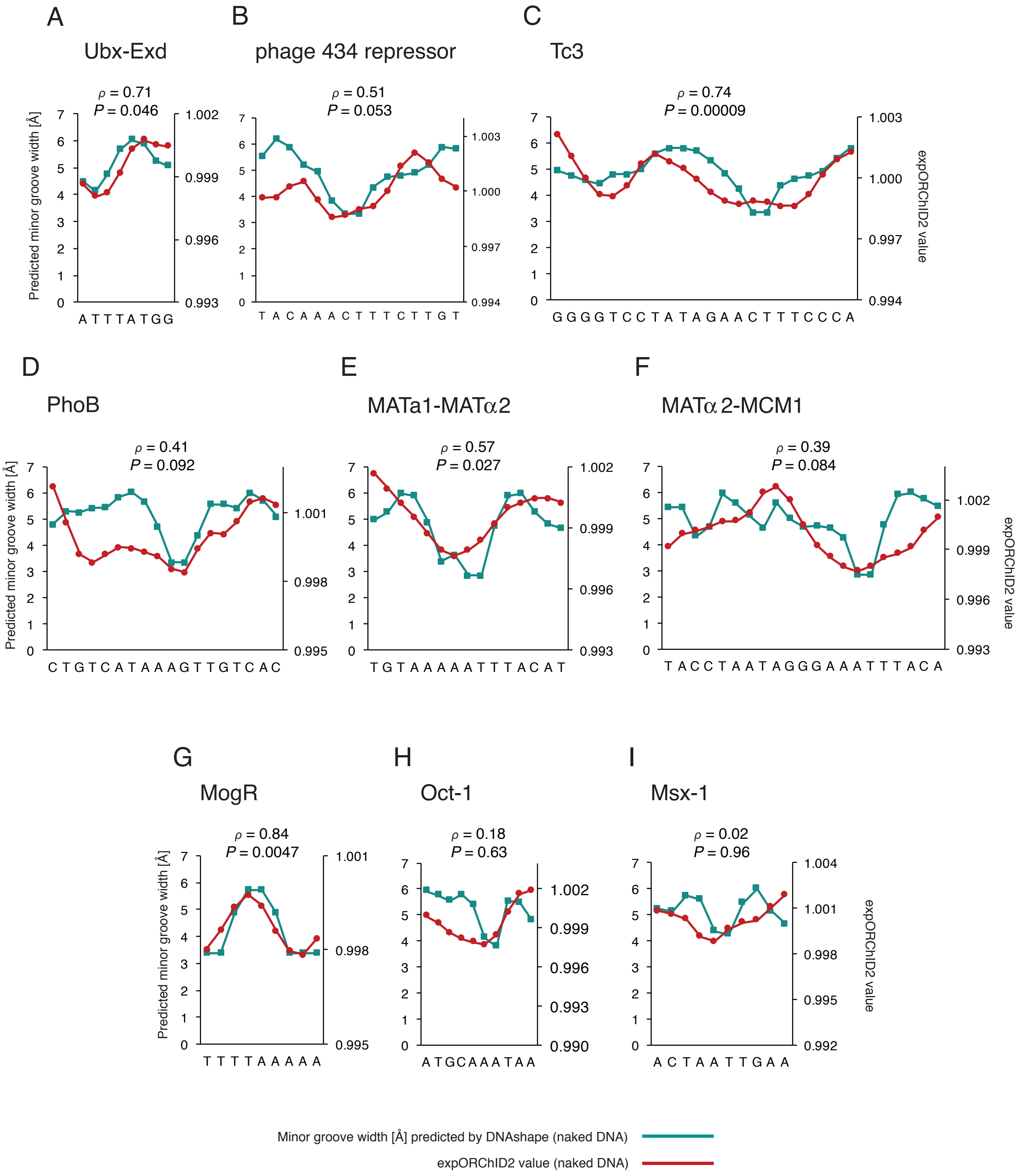
A
Ubx-Exd
ρ = 0.71
P = 0.046
ATTTATGG
B
phage 434 repressor
ρ = 0.51
P = 0.053
TACAAACTTTCTTGT
C
Tc3
ρ = 0.74
P = 0.00009
GGGGTCCTATAGAACTTTCCCA
D
PhoB
ρ = 0.41
P = 0.092
CTGTCATAAAGTTGTCAC
E
MATa1-MATα2
ρ = 0.57
P = 0.027
TGTAAAAATTTACAT
F
MATα2-MCM1
ρ = 0.39
P = 0.084
TACCTAATAGGGAAATTTACA
G
MogR
ρ = 0.84
P = 0.0047
TTTTAAAAA
H
Oct-1
ρ = 0.18
P = 0.63
ATGCAAATAA
I
Msx-1
ρ = 0.02
P = 0.96
ACTAATTGAA
Predicted minor groove width [Å]
expORChID2 value
Minor groove width [Å] predicted by DNAshape (naked DNA)
expORChID2 value (naked DNA)

# Fig. S5

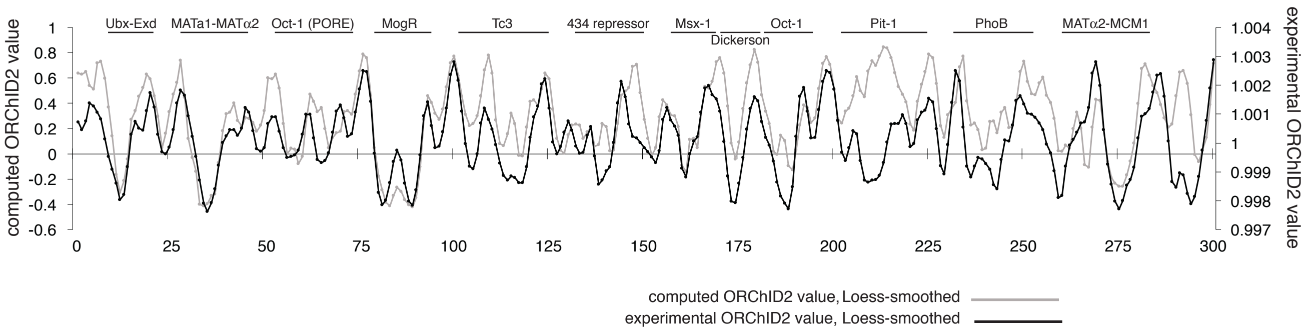

Ubx-Exd
MATa1-MATα2
Oct-1 (PORE)
MogR
Tc3
434 repressor
Msx-1
Dickerson
Oct-1
Pit-1
PhoB
MATα2-MCM1
computed ORChID2 value
experimental ORChID2 value
computed ORChID2 value, Loess-smoothed
experimental ORChID2 value, Loess-smoothed

# Fig. S6

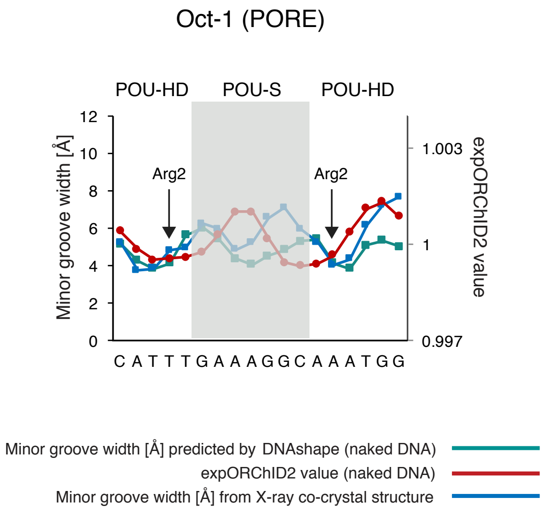
Oct-1 (PORE)
POU-HD
POU-S
POU-HD
Minor groove width [Å]
expORChID2 value
Arg2
Arg2
12
10
8
6
4
2
0
1.003
1
0.997
C A T T T G A A A G G C A A A T G G
Minor groove width [Å] predicted by DNAshape (naked DNA)
expORChID2 value (naked DNA)
Minor groove width [Å] from X-ray co-crystal structure
